# Moderately Hypofractionated Intensity Modulated Radiation Therapy With Simultaneous Integrated Boost for Prostate Cancer: Five-Year Toxicity Results From a Prospective Phase I/II Trial

**DOI:** 10.3389/fonc.2020.01686

**Published:** 2020-08-21

**Authors:** Anthony Ricco, Nitai Mukhopadhyay, Xiaoyan Deng, Diane Holdford, Vicki Skinner, Siddharth Saraiya, Drew Moghanaki, Mitchell S. Anscher, Michael G. Chang

**Affiliations:** ^1^Massey Cancer Center, Department of Radiation Oncology, Virginia Commonwealth University Health System, Richmond, VA, United States; ^2^Department of Biostatistics, Virginia Commonwealth University, Richmond, VA, United States; ^3^Virginia Commonwealth University Health System, Virginia Commonwealth University, Richmond, VA, United States; ^4^Hunter Holmes McGuire Veterans Administration Medical Center, Richmond, VA, United States

**Keywords:** prostate radiation therapy, moderate hypofractionation, patient reported outcome measures, clinical trial, simultaneous integrated boost, pelvic lymph nodes, genitourinary toxicity, gastrointestinal toxicity

## Abstract

**Background:**

In this phase I/II trial, 5-year physician-assessed toxicity and patient reported quality of life data is reported for patients undergoing moderately hypofractionated intensity modulated radiation therapy (IMRT) for prostate cancer using a simultaneous integrated boost (SIB) and pelvic lymph node (LN) coverage.

**Materials and Methods:**

Patients with T1-T2 localized prostate cancer were prospectively enrolled, receiving risk group based coverage of prostate ± seminal vesicles (SVs) ± pelvic lymph nodes (LNs). Low risk (LR) received 69.6 Gy/29 fractions to the prostate, while intermediate risk (IR) and high risk (HR) patients received 72 Gy/30fx to the prostate and 54Gy/30fx to the SVs. If predicted risk of LN involvement >15%, 50.4 Gy/30fx was delivered to pelvic LNs. Androgen deprivation therapy was given to IR and HR patients.

**Results:**

There were 55 patients enrolled and 49 patients evaluable at a median follow up of 60 months. Included were 11 (20%) LR, 23 (41.8%) IR, and 21 (38.2%) HR patients. Pelvic LN treatment was given in 25 patients (51%). Prevalence rates of late grade 2 GI toxicity at 1, 3, and 5 years was 5.8, 3.9, and 5.8%, respectively, with no permanent grade 3 events. Prevalence rates of late grade 2 GU toxicity at 1, 3, and 5 years rates were 15.4, 7.7, and 13.5%, respectively, with three grade 3 events (5.8%). The biochemical relapse free survival at 5 years was 88.3%. There were no local, regional, or distant failures, with all patients still alive at last follow up.

**Conclusion:**

Moderate hypofractionation of localized prostate cancer utilizing a SIB technique and LN coverage produces tolerable acute/late toxicity. Given equivalent efficacy between moderate hypofractionation schedules, the optimal regimen will be determined by long-term toxicity reported from both the physician and patient perspective.

**Clinical Trial Registration:**

www.ClinicalTrials.gov, identifier NCT01117935, Date of Registration: 5/6/2010.

## Introduction

External beam radiation treatment (EBRT) has been a standard treatment in organ confined prostate cancer with high rates of biochemical control and acceptable rates of acute and late toxicity with up to 20-year follow up (1). Advances in the precision and conformality of external beam radiation delivery have allowed dose escalation to decrease biochemical failure rates (BF) while reducing gastrointestinal (GI) and genitourinary (GU) toxicities, in addition to possibly decreasing PCSM (1, 2). These advances occurred during the same time period that radiobiologic models suggested prostate cancer’s would be more sensitive to hypofractionation than conventional fractionation which in turn led to the successful completion of multiple randomized controlled trials comparing standard to moderate hypofractionation. These trials demonstrated equivalent BF, PCSM, overall survival (OS) (3–13). In 2018, this led to a joint ASTRO, ASCO, and AUA guideline recommending moderate hypofractionation be offered across low, intermediate, and HR groups regardless of age, comorbidity, and urinary function (14).

Consensus opinion is that moderate hypofractionation has a similar risk of acute GU and late GU/GI toxicity compared to conventional EBRT with higher rates of acute GI toxicity (14). However, the optimal hypofractionation scheme remains unknown, as there was significant heterogeneity between moderate hypofractionation schedules without one demonstrating superiority. In addition, few trials had included pelvic nodal treatment using a SIB technique (3). Our institutional policy in 2010 was to deliver between 75.6 and 77.4 Gy in 1.8 Gy fractions to the prostate. We opened a phase I/II trial at that time to deliver 69.6 to 72 Gy at 2.4 Gy per fraction in 29 to 30 fractions to the prostate, while elective SV at 1.8 Gy/fx and pelvic nodal coverage at 1.68 G/fx were delivered depending on risk of involvement using an SIB technique.

With similar efficacy between moderate hypofractionation trials, prospectively collected long-term toxicity reported from both the physician and patient perspective will help to distinguish potential optimal regimens. In this manuscript, we provide mature 5 year toxicity data in addition to patient reported outcomes for a moderately hypofractionated schedule utilizing pelvic nodal treatment with SIB technique.

## Methods

### Study Design

This phase I/II single-institutional trial sought to assess the rates of acute and late toxicity with a secondary endpoint of biochemical control with risk-adapted moderately hypofractionated IMRT (ClinicalTrials.gov identifier: XXXX). Eligible patients had clinically node negative adenocarcinomas of the prostate, stage T1–T3, with KPS ≥ 80 (15). Prior to enrollment, patients underwent pretreatment evaluation with history and physical, digital rectal examination, complete blood count, and liver function tests. Serum prostate-specific antigen (PSA) and prostatic biopsies were required within 12 months of enrollment. Patients also had pre-study, post-study, and weekly during treatment assessments of GI, GU, and erectile function which were both physician and patient assessed. Patients were stratified into LR, IR, and HR groups according to National Comprehensive Cancer Network (NCCN, version 1.2010) guidelines, with HR patients receiving a CT scan of the abdomen and pelvis and Tc99m-MDP bone scans to rule out metastatic disease. Patients were excluded if they had previous history of malignancy other than skin cancer within 5 years of treatment, had a history of inflammatory bowel disease or collagen vascular disease, or had prior pelvic radiotherapy for any reason.

Low risk patients received 69.6 Gy to the prostate alone in 29 fractions of 2.4 Gy each. The IR and HR patients received 72 Gy to the prostate in 30 fractions of 2.4 Gy each and 54 Gy in 30 fractions of 1.8 Gy fractions to the proximal 1 cm SV using dynamic IMRT and a simultaneously integrated boost (SIB) technique. Patients received pelvic nodal irradiation to 50.4 Gy in 30 fractions of 1.68 Gy with SIB technique if their calculated Roach formula risk of LN involvement *geq* 15% (16). Androgen deprivation therapy was given 2 months prior to the initiation of radiotherapy for a total duration of 6 months to IR patients and 36 months to HR patients (17, 18).

Intraprostatic fiducial markers were placed transperineally in all patients for daily image guidance. CT simulation images were captured from the L1 vertebral body superiorly to mid-thigh inferiorly with 3-mm slice thickness. Patients were instructed to maintain a full bladder and empty rectum for simulation and daily treatments. The prostate was defined as the GTV and CTV; the proximal 1 cm of SV was contoured separately. CTV to PTV expansion was 7 mm in all directions except for 3 mm posteriorly. Pelvic nodal PTV was defined as a 10 mm expansion in all directions off external, internal, and common iliac vessel contours.

Organs at risk (OAR) included rectum, posterior rectum, bladder, small bowel, femoral heads, and skin contoured according to national guidelines, with dose constraints listed in Table 1 (19). Rectum was contoured from the anterior flexion of the recto-sigmoid superiorly to the level of the ischial tuberosity inferiorly. Posterior rectum consisted of the posterior half of the rectum on each axial CT slice separating the midway point between the anterior-most and posterior-most aspects of the rectal contour on each axial CT slice. A seven-field IMRT beam arrangement was used on all patients. Daily image guidance with kilovoltage or megavoltage orthogonal imaging, Calypso beacons (Varian, Palo Alto, CA, United States), or cone beam CT was performed on all patients before treatment. An orthogonal portal-image pair is observed immediately prior to initiating the treatment sequence. The treatment iso-center is positioned relative to internal radioopaque markers based upon the CT-directed marker position recorded on the first day of the IMRT. The couch was repositioned when prostate fiducial markers were greater than 2 mm from their initial positions, and portal imaging was repeated with markers were greater than 5 mm from their original positions.

**TABLE 1:** Target and normal tissue dose constraints.

Target tissue	Dose (Gy)	Goal ≥ (%)	Protocol violation %
Prostate PTV – Low risk	69.6/29 Fx	95	<90
Prostate PTV – Intermediate/high risk	72/30 Fx	95	<90
Seminal vesicle PTV	54/30 Fx	95	<90
Lymph node PTV	50.4/30 Fx	95	<90
**Organ at risk**	Dose (Gy)	Goal ≤ (%)	≥(%)
Rectum	36	50	60
Rectum	54	30	40
Rectum	66	20	30
Rectum	72	5	15
Rectum	70	<10 cc	>12 cc
Posterior half of rectum	45	2	12
Small bowel	25	50	60
Small bowel	45	33	43
Small bowel	52	2	12
Bladder	50	50	60
Bladder	64	25	35
Bladder	72	3	13
Non-PTV bladder	72	3	13
Femoral heads	35	50	60
Femoral heads	40	10	20
Femoral heads	45	2	12
Skin 1 cm	30	50	60
Skin 1 cm	35	10	20
Skin 1 cm	45	2	12

### Toxicity and Analysis

The primary endpoint of this phase I/II study was to measure the physician-reported cumulative rate of late grade 2 or higher GI and GU toxicity. Secondary endpoints included acute GI/GU toxicity, transient late GI/GU toxicity, prevalence of late GI/GU toxicity at last follow up, patient reported toxicity/quality of life indicators, and bRFS. Acute toxicity was defined as onset and resolution within 90 days of treatment. Late toxicity was defined as unresolved acute toxicity or onset beyond 90 days, with patients requiring medication at or beyond 90 days defined as having late grade 2 GU toxicity. A historic rate of grade 2 toxicity of 14.4% was utilized for comparison, with a rate of 30% or higher deemed as unacceptable toxicity per protocol. The Data and Safety Monitoring Committee would stop the protocol if the rate of late grade 3 or higher GI or GU toxicity was greater than 7%. The study aimed to accrue 55 patients for an 86% power utilizing a 1-sample, 1-sided Fisher exact test at the 5% level of significance allowing for a 10% dropout rate.

The physician-reported Common Terminology Criteria for Adverse Events (CTCAE, version 3.0), the patient-reported International Prostate Symptom Score (IPSS), and the patient-reported International Index of Erectile Function 5 (IIEF) represented the data collection mechanisms of monitoring acute and late GI and GU toxicity (20–22). Toxicity was assessed prior to EBRT as baseline assessments, weekly during EBRT, and at every follow up visit. Follow up schedule included initial visit 4–6 weeks after treatment, every 4 months for 3 years, and then semi-annually for 2 years. All analyses were performed in statistical software R v3.2.1 (R Foundation for Statistical Computing, Vienna, Austria).

## Results

Between June 2010 and June 2013, 55 patients were enrolled and data from 52 patients were available for the analyses. Two patients were enrolled and then withdrew consent before receiving any radiotherapy. The final patient developed grade 3 diarrhea felt to be infectious in nature midway through treatment, and discontinued therapy at that time. Three of the 52 patients who completed radiotherapy did not complete the minimum of 12 months of follow up which left 49 patients evaluable for biochemical recurrence and toxicity. The median follow up of this group was 60 months (range 8–60 months), as all patients were followed only to a maximum of 60 months.

Patient demographics and characteristics can be found in Table 2. Eleven patients had LR disease, 18 had IR disease with nodal risk <15%, 5 had IR disease with nodal risk ≥ 15%, and 21 had HR disease. Twenty-five patients received prostate and pelvic nodal treatment with SIB technique when indicated per protocol by Roach Formula result, with 24 receiving prostate-only treatment. Ultimately, 44 out of 55 (80%) patients enrolled received ADT.

**TABLE 2:** Patient Characteristics*.

Variable	Number of patients (percentage)
Patients evaluable	52
Age	65 (51–80)
**Race**	
African American	28 (53.8%)
White (Non-hispanic)	24 (46.2%)
**Gleason Score**	
6	14 (27.5%)
7 (3 + 4)	17
7 (4 + 3)	2
8	9 (17.7%)
9	9 (17.7%)
10	1 (2.0%)
**Clinical T stage**	
T1	40
T2a or T2b	7
T2c	1
T3	3
T4	0
PSA	
iPSA*	6.6 (2–147)
iPSA ≤10	31 (59.6%)
iPSA >10, ≤20	12 (23.1%)
iPSA >20	9 (17.3%)
**Risk classification**	
Low	11 (20%)
Intermediate	23 (41.8%)
High	21 (38.2%)
**ADT**	
Yes	44 (80%)
No	11 (20%)

*iPSA, initial prostate-specific antigen at initiation of treatment, *median age in years (range).

### Physician-Reported Toxicity

**FIGURE 1:**
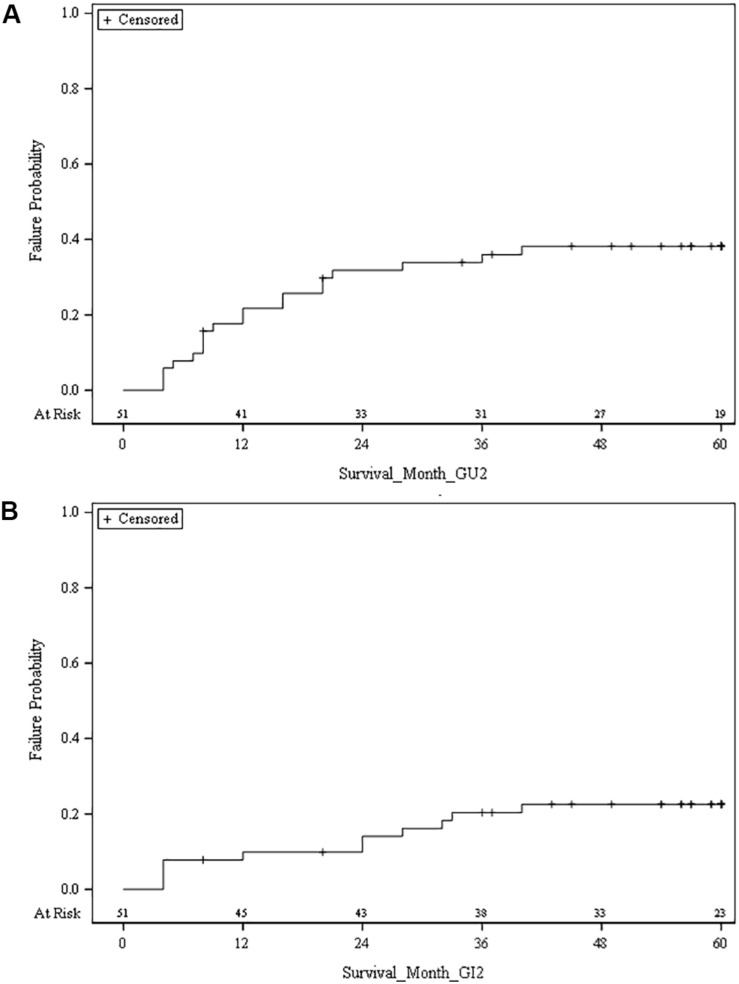
**(A)** Cumulative risk of grade 2 or higher late genitourinary (GU) toxicity. **(B)** Cumulative risk of grade 2 or higher late gastrointestinal (GI) toxicity.

**FIGURE 2:**
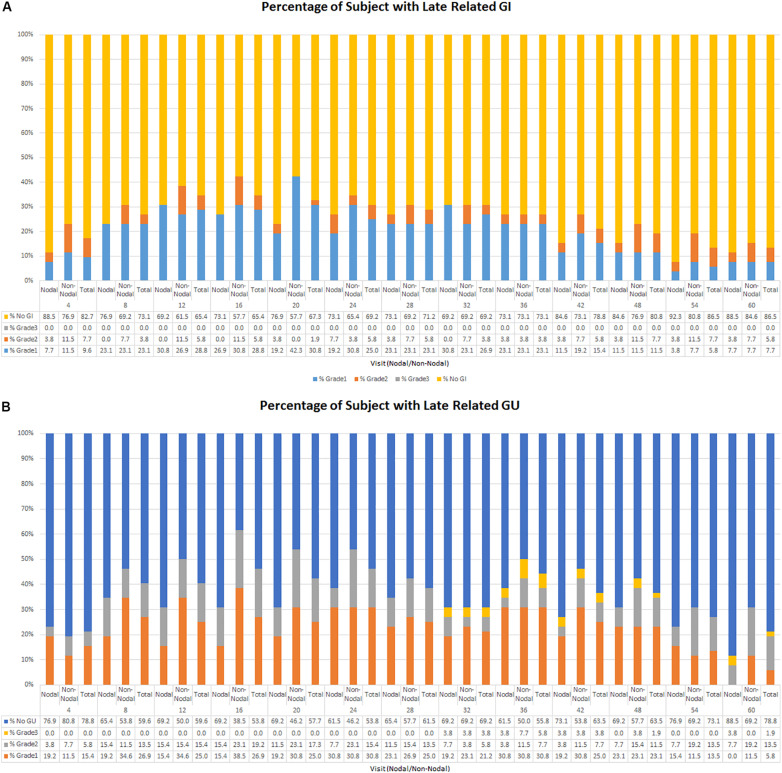
**(A)** Percentage of late GI related toxicity by grade. **(B)** Percentage of late GU related toxicity by grade.

Late GI toxicity consisted mostly of self-limited grade 1/2 toxicities which resolved over time. At 5 years, the cumulative incidence of late grade 2 or higher GI toxicity was 22.6% (Figure 1). The percentage of patients experiencing a late grade 2 GI toxicity at 1, 3, and 5 years was 5.8, 3.8, and 5.8%, respectively (Figure 2). Of the 12 patients who were found to have late GI toxicities of grade 2 or higher, 1 reported anal incontinence, 5 reported flatulence, 4 reported diarrhea, and 2 reported either rectal bleeding or proctitis. There were no patients with unresolved late permanent grade 3 GI toxicity at last follow up. Late grade 2 or higher GI toxicities that did not resolve by 60 months included 3 out of 52 patients (5.8%); 1 reported flatulence, 1 reported anal incontinence, and 1 reported diarrhea. All three of these toxicities did resolve with subsequent follow up at the 74, 61, and 67 months timepoint after treatment, respectively.

At 5 years, the cumulative incidence of late grade 2 or higher GU toxicity was 38.2% (Figure 1). The percentage of patients experiencing a late grade 1 GU toxicity at 1, 3, and 5 years was 25, 30.1, and 5.8%, respectively, with grade 2 rates at 15.4, 7.7, and 13.5%, respectively (Figure 2). There were 3 patients (5.8%) who experienced grade 3 GU toxicity, which included bladder spasms, urinary incontinence, and cystitis. There were 4 (7.7%) late grade 2 or higher toxicities which did not resolve by 60 months; 1 with radiation cystitis, 1 with urethral stricture, 1 with urinary retention, and 1 unknown which all resolved at the 74, 61, 68, and 61 months timepoint after treatment, respectively.

In the subset of patients who received pelvic nodal radiation therapy when indicated per protocol by Roach Formula result, there was a trend toward lower rates of late GU and GI toxicity, which can be seen in Figure 2. For example, the prevalence of any late GI toxicity at 1, 3, and 5 years was 42.3% vs. 30.8%, 26.9% vs. 26.9%, and 15.4% vs. 11.5% for non-nodal treatment vs. nodal treatment, respectively. In addition, the prevalence of any late GU toxicity at 1, 3, and 5 years was 51.8% vs. 30.8%, 42.3% vs. 34.6%, and 30.7% vs. 7.7% for non-nodal treatment vs. nodal treatment, respectively. There were no statistically significant differences in cumulative rates of late GI or GU toxicity between patients who did and did not receive pelvic nodal radiation therapy with SIB technique. Acute toxicity maximum grade stratified by nodal and non-nodal treatments can be seen in Table 3.

**TABLE 3:** Maximum acute toxicity grade stratified by nodal and non-nodal treatment groups.

Number of subjects with related acute toxicity by grade					
Group	Grade				
		1	2	3	Total
Nodal (*N* = 26)	#	5	18	3	26
	%	19.23	69.23	11.54	100
Non-nodal (*N* = 26)	#	8	16	1	25
	%	32	64	4	100
Total		**13**	**34**	**4**	**51**

### Patient Reported Toxicity/Quality of Life Indicators

**FIGURE 3:**
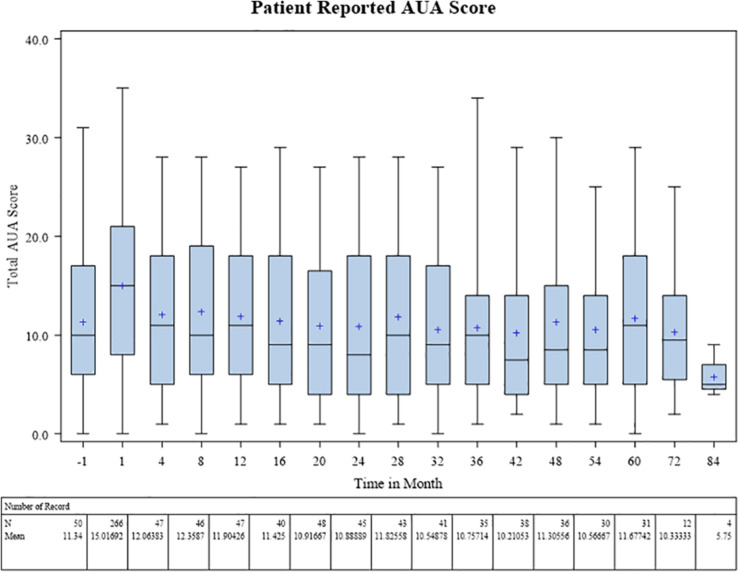
Patient reported AUA score.

Baseline patient-reported IPSS score was on average 11.3 prior to radiotherapy. During treatment this rose to a mean of 15.0, and subsequently fell back to pretreatment baseline at 1 year with an average score of 11.9. The 3 and 5 year IPSS scores remained at baseline, 10.8 and 11.7, respectively, on average (Figure 3).

**FIGURE 4:**
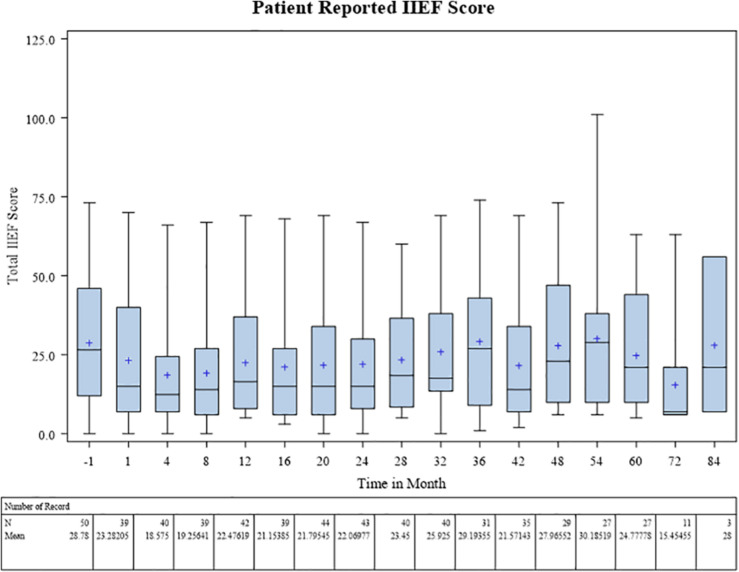
Patient reported IIEF score.

Baseline patient-reported IIEF score was on average 28.8 prior to radiotherapy. During treatment this fell to a mean 18.6 at 4 months post treatment coinciding with ADT use. Scores increased gradually over time to near baseline levels (24.8) by 5 years (Figure 4).

### Tumor Control

**FIGURE 5:**
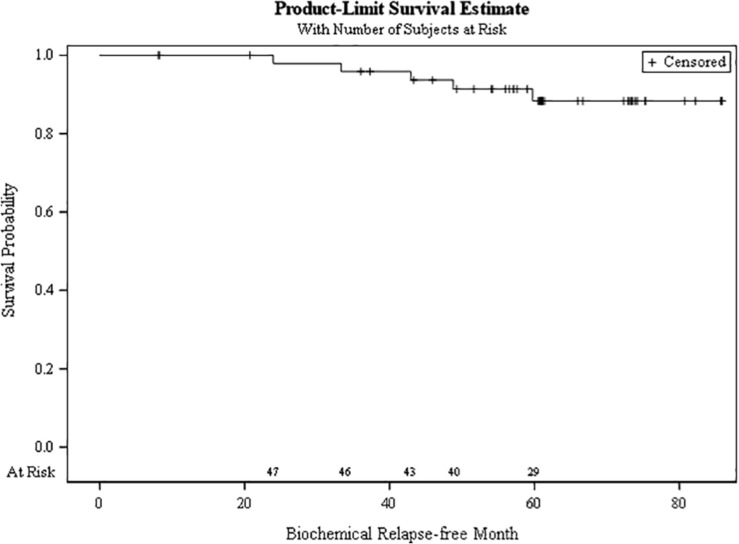
Biochemical relapse free survival at 5 years.

The bRFS at 5 years for the cohort was 88.3% (Figure 5). There were no local, regional, or distant failures, with all patients still alive at last follow up.

## Discussion

Moderate hypofractionation in prostate cancer has now been accepted and recommended to be offered across all risk groups (14). There remains uncertainty, however, about the optimal dose-fractionation, as significant heterogeneity among moderate hypofractionation trials exist. In addition, many trials’ toxicity outcomes are difficult to interpret (3). For example, both the PROFIT and CHHiP trials show no difference in late GI or GU toxicity between conventional and moderate hypofractionation arms, however, the RTOG 0415 trial showed a significant difference and the MD Anderson trial showed trend toward worse late GI toxicity in the hypofractionated arms (4, 5, 10, 12). This current prospective clinical trial adds to the data on toxicity rates with moderate hypofractionation, strengthened by the inclusion of hypofractionated pelvic nodal treatments in a subset of patients utilizing modern radiotherapy techniques with IMRT using an SIB technique and daily image guidance which has only recently been introduced into routine clinical care. As moderate hypofractionation’s efficacy in localized prostate cancer treatment has now been accepted, prospectively collected long-term toxicity and patient quality of life data will help to distinguish the optimal dose fractionation schedule.

With a median follow up of 5 years, the updated toxicity data from this study once again demonstrates physician and patient-reported late GI and GU toxicity rates that are comparable to similar series. The vast majority of patients experienced transient grade 2 or higher late GI and GU toxicities which resolved over time. While the physician-reported GU toxicity exceeded the upper limit of tolerance by trial design, there were only 7.7% late grade 2 or higher toxicities (four patients) and 5.8% of late grade 2 or higher toxicities (three patients) which did not resolve by 5 years. The grade 3 GU toxicity rate was 5.8%, comparable to other hypofractionation trials such as 3.5% on RTOG 0415 and 0.7% in the PROFIT trial (4, 10). Neither the GU nor GI toxicity rates increased significantly between 2 and 5 years post-treatment, lending evidence for long term safety. However, it is clear that even at 5 years of follow up, there are grade 2 toxicities (5.8% GI and 7.7% GU) which take longer to resolve; in our study up to 74 months from treatment. Further reporting of toxicity rates with more than 5 years of follow up are warranted.

A unique feature of the current trial is its inclusion of pelvic nodal treatment in a subset of patients utilizing a SIB technique. While this technique for treating pelvic nodes was first pioneered in the RTOG 0529 trial in 2005 in the setting of anal cancer (23), our trial was one of few to include SIB of pelvic nodes in the prostate cancer setting and is one of only three trials so far with median follow up of 5 years or longer (24–26). It would be expected to see higher rates of late GI toxicity in the nodal treatment cohort, however, we instead saw a trend toward less toxicity. In addition, we saw a trend toward lower rates of late GU toxicity in the nodal treatment arm. This is largely a spurious finding in the current trial, driven by small sample size and a select number of patients in the non-nodal treatment group which consistently demonstrated GI and GU toxicity over multiple time points. At the least, this toxicity data indicates that SIB treatment of pelvic lymph nodes in prostate cancer appears safe. Although the benefit of pelvic nodal treatment has yet to be established, it may play a larger role in the management of prostate cancer as recent RTOG clinical trials continue to accrue and completed trials’ data mature (27, 28).

The largest phase III trial to include pelvic nodal treatment in testing moderate hypofractionation was the Fox Chase Cancer Center (FCCC) study, which treated 31.7% of its patients with pelvic nodal fields (9, 25). 151 patients were enrolled on the hypofractionation arm with a higher proportion of HR patients and thus ADT use than the MDACC trial (14.6% LR, 57% IR, 28.5% HR, and 45% ADT), but still less use than the current trial (80%). The pelvis was treated to 50–52 Gy in 26 fractions at 1.92 Gy per fraction and 70.2 Gy in 26 fractions of 2.7 Gy to the prostate. PTV expansions in the hypofractionation arm was 7 mm in all directions except for 3 mm posteriorly, the same as the current protocol. Updated results show no significant difference in any QOL measures using EPIC, IPSS, and EQD5 scores, but interestingly a trend toward a worse rate of distant metastases at 10 years in the hypofractionation arm (9, 29).

Di Muzio et al. also reported 5 year results of a large phase I/II trial involving pelvic lymph node coverage in intermediate and HR patients (24). Fifty-three IR and 80 HR patients received 74.2 Gy in 28 fractions of 2.65 Gy and 51.8 Gy at 1.85 Gy per fraction to the pelvic lymph nodes. PTV expansion was 10-mm in all directions except 8 mm posteriorly. The entire SVs were covered in all patients, with a risk adapted approach to dose. ADT was used in 70% of patients. Cumulative rates of grade 2 and 3 GU toxicities were 20.2 and 5.9%, respectively, with cumulative rates of grade 2 and 3 GI toxicities 17 and 6.3%, respectively. While our rates of GU toxicities are comparable, our rate of GI toxicity is numerically lower and could be explained by larger PTV expansions or complete SV coverage in the Di Muzio trial. In addition, there have been other smaller trials confirming that SIB treatment of pelvic lymph nodes is safe, at various fraction sizes from 1.56 Gy to 2 Gy (total doses from 50 Gy to 56 Gy) with all trials showing acute grade 3 or higher GI toxicity <3% and late grade 3 or higher GI toxicity <10% (26, 30–36).

We must exercise caution when attempting to identify the optimal regimen of moderate hypofractionation. Given the considerable heterogeneity in dose-fractionation it is difficult to compare toxicity across trials. There was heterogeneity among the control arms of these trials, with some using non-dose-escalated radiotherapy (37, 38) and others using dose-escalated radiotherapy (4–12, 29, 39). Trials were also designed differently, varying between dose-fractionation schemes to be isoeffective to late tissue effects versus dose-escalated or non-dose-escalated, complicating late toxicity analysis. Trials differed in PTV margins, extent of SV coverage in CTV, and ADT use, all of which in a recent meta-analysis have been shown to be significantly correlated with worse acute and late GI toxicity (3). In addition, there were differences in quality of life instruments, definitions for assigning grade 2 GI or GU toxicity, image guidance, and dose constraints.

Biologically effective doses also varied widely between hypofractionation trials, from 156 to 211 Gy assuming an alpha/beta ratio of 1.5, and between 85 to 108 Gy assuming an alpha/beta ratio of 5, which are values typical for prostate tumor control and for late rectal toxicity, respectively (40–46). Brenner and Hall discuss that the trials with the highest BED_1_._5_ also saw significant late GI and GU toxicity, and that BED_1_._5_ for moderately hypofractionated radiotherapy should not exceed 183 Gy (46). The HYPRO trial had the highest BED_1_._5_ (211 Gy) prescribed and saw increased cumulative rates of late grade 3 or higher GU toxicity higher in the hypofractionation arm (19.0% vs. 12.9%), with toxicity non-inferiority unable to be confirmed (47). RTOG 0415 (BED_1_._5_ 186 Gy), the second highest value, also showed higher late grade 2 or 3 GI and GU toxicity (HR 1.31–1.59) (10). The CHHiP and PROFIT trials used the lowest BED_1_._5_ of moderate hypofractionation trials (BED_1_._5_ 180) and both saw no increased risk of late GI or GU toxicity (4, 5).

Our trial shares commonalities amongst other moderate hypofractionation trials. The MD Anderson Cancer Center (MDACC) trial utilized the same fraction as IR and HR patients on our study, receiving 72 Gy to the prostate in 30 fractions of 2.4 Gy each at a BED of 187 Gy (12). This trial had 102 patients on their hypofractionation arm utilizing IMRT technique, consisting of 27% low, 72% intermediate, and 1% HR. Only the prostate and proximal SV was treated. The protocol mandated that PTV expansion was 10 to 15 mm in all dimensions except for 4 to 8 mm posteriorly, compared to 7 mm in all directions except for 3 mm posteriorly in the current protocol. Approximately 20% of patients received ADT, compared to 80% on our trial. Late GU (grade 2 or 3) toxicity was no different between arms, with 16.4% in the conventional arm and 15.1% in the hypofractionated arm at 8 years. There was a trend (*p* = 0.08) toward worse late (grade 2 or 3) GI toxicity in the moderate hypofractionation arm, up to 12.6% compared to 5% at 8 years. This however, could be reduced to 8.6% if V65 < 15%. These values compare favorably to our grade 2 or higher GI toxicity (5.8%) and GU toxicity (7.7%) at 5 years.

The current clinical trial is ultimately limited by multiple factors, including its phase I/II design and low patient numbers, highlighting the need for large prospectively followed populations. In addition, we did not utilize a patient-reported quality of life instrument for GI toxicity. Due to sample size, we were unable to make specific conclusions about toxicity differences between pelvic nodal and prostate only patients. Like most moderate hypofractionation trials, our follow up is limited to 5 years at present and further follow-up is needed for evaluation of potential late effects.

## Conclusion

The results from this trial add to the growing body of maturing data with 5 or more years of follow up with moderately hypofractionated courses of prostate radiotherapy. The data demonstrate similar rates of late toxicity with or without pelvic nodal irradiation using a SIB technique. The use of 2.4 Gy/day to the prostate also produces similar biochemical control and acceptable acute/late toxicity when compared to standard fractionation, justifying its appropriateness for routine use in clinical practice.

## Data Availability Statement

The raw data supporting the conclusions of this article will be made available by the authors, without undue reservation.

## Ethics Statement

The studies involving human participants were reviewed and approved by the Virginia Commonwealth University IRB and Hunter Holmes McGuire Veterans Affairs Hospital IRB. The patients/participants provided their written informed consent to participate in this study.

## Author Contributions

MC, MA, and DM conceived of the project and worked toward securing IRB approval and funding. MC, DH, and VS performed the data-collection. NM and XD performed the statistical analysis. AR wrote the initial draft of the manuscript. SS, DM, MA, and MC provided editorial input. All authors contributed to the article and approved the submitted version.

## Conflict of Interest

The authors declare that the research was conducted in the absence of any commercial or financial relationships that could be construed as a potential conflict of interest.

## Abbreviations

ADT: androgen deprivation therapy
ASCO: American Society of Clinical Oncology
ASTRO: American Society for Radiation Oncology
AUA: American Urological Association
BED: biologically effective dose
bRFS: biochemical relapse free survival
CT: computed tomography
CTCAE: Common Terminology Criteria for Adverse Events
CTV: clinical target volume
EBRT: external beam radiation therapy
GI: gastrointestinal
GU: genitourinary
HR: high risk
IIEF: international index of erectile function
IMRT: intensity modulated radiation therapy
IPSS: International Prostate Symptoms Score
IR: intermediate risk
KPS: Karnofsky performance status
LN: lymph node
LR: low risk
NCCN: National Comprehensive Cancer Network
OAR: organ at risk
PCSM: prostate cancer specific mortality
PSA: prostate specific antigen
PTV: planning target volume
SIB: simultaneous integrated boost
SV: seminal vesicle.
